# Shrimp miR-965 transfers tumoricidal mitochondria

**DOI:** 10.1186/s12575-022-00178-8

**Published:** 2022-10-26

**Authors:** Hyueyun Kim, Ji Ha Choi, Chang Mo Moon, Jihee Lee Kang, Minna Woo, Minsuk Kim

**Affiliations:** 1 Department of Pharmacology, College of Medicine, Ewha Womans University, Magokdong-Ro 2-Gil, Gangseogu, Seoul, 07804 Republic of Korea; 2 Department of Internal Medicine, College of Medicine, Ewha Womans University, Seoul, Republic of Korea; 3 Department of Physiology and Tissue Injury Defense Research Center, College of Medicine, Ewha Womans University, Seoul, Republic of Korea; 4 Toronto General Hospital Research Institute and Division of Endocrinology and Metabolism, Department of Medicine, University Health Network, University of Toronto, Toronto, ON Canada

**Keywords:** Mitochondria, Microfluidics, Tunneling nanotube, Breast tumor, Cotton candy, Tomographic microscope, Refractive index

## Abstract

**Background:**

Micro RNA of *Marsupenaeus japonicas* has been known to promote apoptosis of tumor cells. However, the detailed mechanisms are not well understood.

**Results:**

Using tomographic microscope, which can detect the internal structure of cells, we observed breast tumor cells following treatment of the miRNA. Intriguingly, we found that mitochondria migrate to an adjacent tumor cells through a tunneling nanotube. To recapitulate this process, we engineered a microfluidic device through which mitochondria were transferred. We show that this mitochondrial transfer process released endonuclease G (Endo G) into tumor cells, which we referred to herein as unsealed mitochondria. Importantly, Endo G depleted mitochondria alone did not have tumoricidal effects. Moreover, unsealed mitochondria had synergistic apoptotic effects with subtoxic dose of doxorubicin thereby mitigating cardiotoxicity.

**Conclusions:**

Together, we show that the mitochondrial transfer through microfluidics can provide potential novel strategies towards tumor cell death.

**Supplementary Information:**

The online version contains supplementary material available at 10.1186/s12575-022-00178-8.

## Introduction

Tunneling nanotubes are thin and long membrane elongations between cells that mediate trafficking of subcellular vesicles, proteins, and organelles [1]. They consist of filamentous-actin and are about 0.05–1 µm in width and 100 µm in length [1] which has been implicated as one mechanism by which mitochondria can be transferred from one cell to another [2]. The speed of mitochondrial migration is about 80–1400 nm/s through tunneling nanotubes [3]. Several studies have been conducted on mitochondrial migration between cells [4–7]. An in vitro study showed that oxidative stress-induced apoptosis of endothelial or H9c2 cardiomyocytes was abolished by transferring of mitochondria from mesenchymal stem cells [8, 9]. Another in vivo study showed that mitochondrial transfer inhibited the hypoxia-based apoptosis in cardiomyocytes [10]. Recent studies suggest that mitochondria can be transferred to support the survival of metabolically compromised cells [11, 12]. Moreover, it has been reported that cancer cells can hijack mitochondria from immune cells via physical nanotubes [13]. Therefore, mitochondrial transfer through tunneling nanotubes can provide important clues in our understanding of tumor cell fate.

We describe here the application of optical tomography whereby a remote imaging technique is used to obtain cross-sections of cells [14]. In optical tomography, projected images are obtained by waves passing through the cells at various angles, and digital images are subsequently reconstructed to obtain the internal structure of cells in a cross-sectional manner [14, 15]. Since the tomographic microscope can reconstruct cell images according to varying refractive index, transparent objects such as lipid droplets and mitochondria can be detected without staining [16, 17].

In this study, we found extracellular vesicles and microRNAs (miRNA) from shell membrane of *Marsupenaeus japonicas*. A miRNA is a small non-coding RNA molecule containing about 22 nucleotides that mediates RNA silencing and posttranscriptional regulation of gene expression [18, 19]. A miRNA can be released into extracellular space and taken up by neighboring cells [20]. From miRNA profiling studies, multiple miRNAs, including miR-6491, miR-6492, miR-6493, miR-6494, or miR-965 have been reported from *Marsupenaeus japonicas* [21, 22]. One of these, miR-965 has been shown to decrease the expression of myeloid cell leukemia-1 (Mcl-1), which blocks the secretion of cytochrome C from mitochondria [22–24].

Here, we examined breast tumor cells and their response to miR-965 and its antagomir by tomographic microscopy. Intriguingly, we found that mitochondria within tumor cells transferred into neighboring tumor cells via tunneling nanotubes and induced apoptosis. Therefore, we hypothesized that the mitochondria of tunneling nanotubes can be effective novel strategy towards tumor cell death. To this end, we conducted experiments on the mitochondrial mechanisms of tumor apoptosis following miR-965 treatment.

## Results

### Crustacean miRNA induces apoptosis of breast tumor cells

**Fig. 1:**
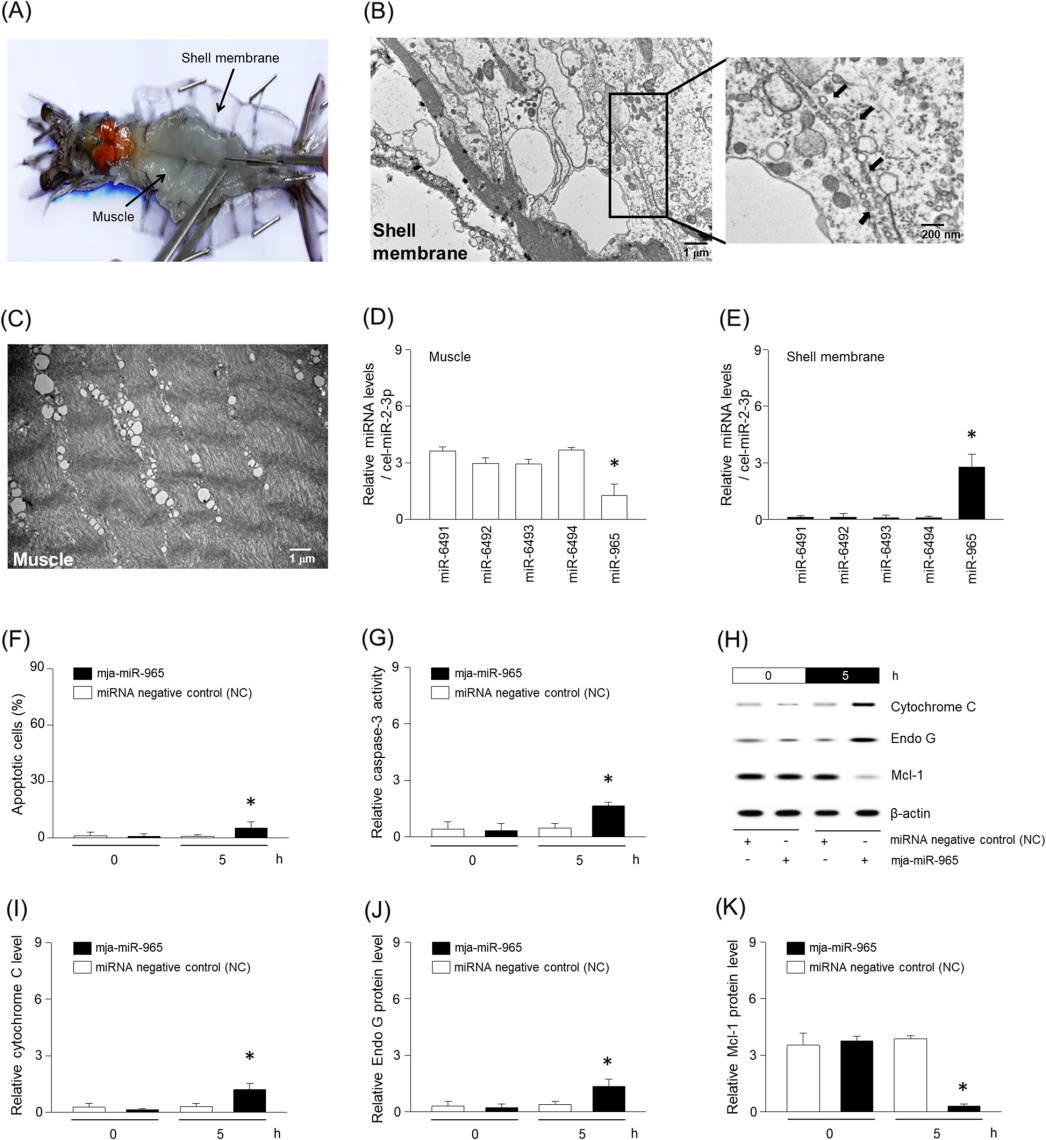
Small size vesicles in abdominal shell membrane of *Marsupenaeus japonicas*. (**A**) Photograph of abdominal shell membrane and muscle from dissected *Marsupenaeus japonicas*. Ultrastructure of (**B**) shell membrane showing small size vesicles (arrows) and (**C**) abdominal muscle. The muscle (**D**) and shell membrane (**E**) were pulverized with a mortar and miRNA microarray determined. Apoptosis (**F**) and cleaved caspase-3 (**G**) were determined using ELISA kit. (**H–K**) Proteins were extracted from MDA-MB-453 cells and expression of cytochrome C, Endo G, or Mcl-1 was determined using western blotting. Results are the means ± SE of 6 experiments in each group. *Significantly different from all other groups, *P* < 0.05

**Fig. 2:**
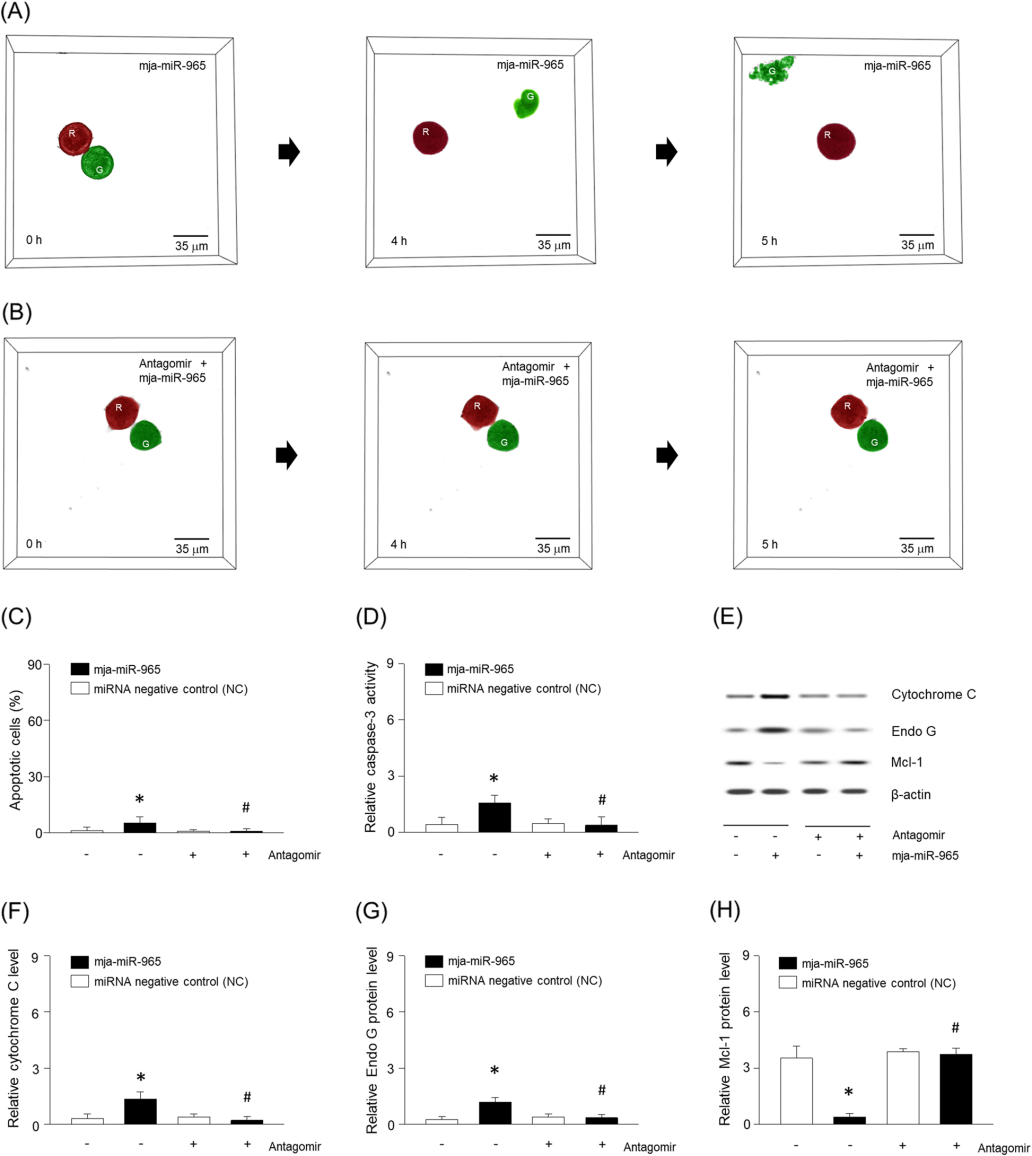
Apoptosis of MDA-MB-453 in response to crustacean miRNA. Following treatment with (**A**) synthetic miR-965 or (**B**) its antagomir, cell images were 3D rendered with top views by tomographic microscopy at 0, 4, or 5 h. Adjacent cells were digitally stained in green or red. Cells were fixed up to 5 h and apoptosis (**C**) and caspase-3 (**D**) were determined using ELISA kit. (**E–H**) Proteins were extracted from MDA-MB-453 cells and expression of cytochrome C, Endo G, or Mcl-1 was determined using western blotting. Results are the means ± SE of 6 experiments in each group. *Significantly different from treatment of miRNA negative control (NC), *P* < 0.05. ^#^Significantly different from treatment of miR-965, *P* < 0.05

The shell membrane of *Marsupenaeus japonicas* (Fig. 1A), contained a number of microvesicles which were readily visible by electron microscopy (Fig. 1B). In contrast, no microvesicles were visible in the isolated muscle (Fig. 1C), through various miRNAs were measured except for mja-miR-965 (Fig. 1D). Intriguingly, mja-miR-965 constituted the predominant fraction in the shell membrane (Fig. 1E). As the shell of crustaceans or miR-965 have been shown to inhibit tumor growth [22, 24–26], we tested the effects of synthetic miR-965 on MDA-MB-453 breast cancer cells and found this to increase apoptosis to 10% of tumor cells compared to less than 0.8% in control group as assessed by ELISA to detect ssDNA (Fig. 1F). Similarly, miR-965 increased cleaved caspase-3, cytochrome C and Endo G (Fig. 1G-J), whereas Mcl-1 levels were decelerated (Fig. 1K). To further visualize these MDA-MB-453 cells, they were scanned with a λ = 520 nm laser beam of tomographic microscopy and the holographic images were recorded and shown as 3D rendering (Fig. 2A and B, Supplementary Fig. 1A-C). Next, we exposed miR-965 or antagomir-965 (antisense of miR-965) to the tumor cells. For easy identification, adjacent cells were digitally colored in red or green and named R and G. After 4 h of exposure with miR-965, G cells were detached and suspended in cell media, which showed morphological alterations such as cell shrinkage and membrane blebbing (Fig. 2A). However, these morphological changes did not occur following treatment with the antagomir (Fig. 2B). The miR-965 again increased apoptosis, as showing by increased cleaved caspase-3, cytochrome C, and Endo G (Fig. 2C-G) with decreased levels of Mcl-1 (Fig. 2H) and its antagomir attenuated the miRNA-induced apoptotic changes.

### Physical mimics of tunneling nanotubes in breast tumor cells

**Fig. 3:**
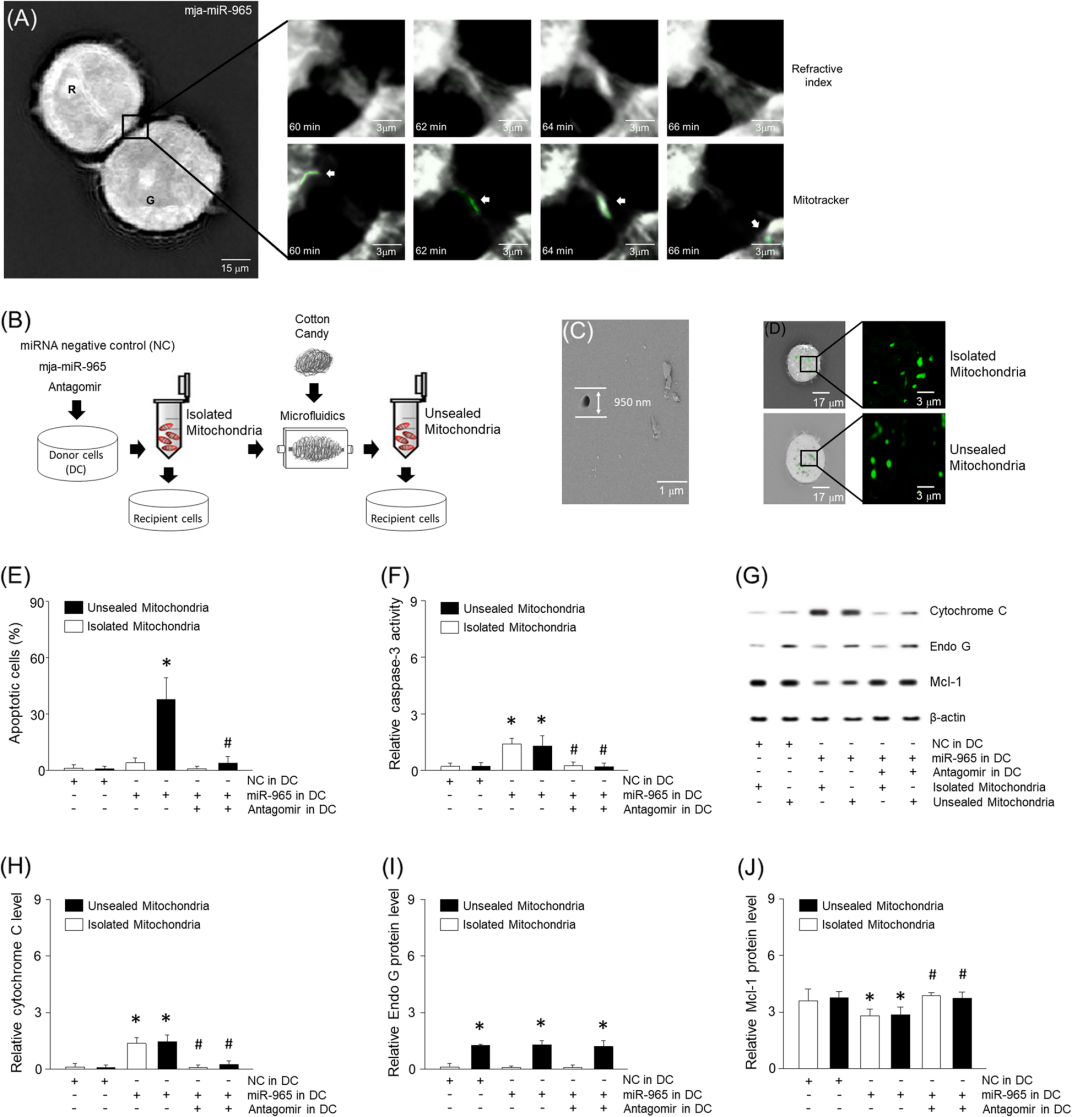
Mitochondrial transport through tunneling nanotubes or narrow microfluidic system. (**A**) Following treatment of synthetic miR-965, refractive index images of MDA-MB-453 were enlarged at 60, 62, 64, or 66 min. Mitochondria were stained with mitotracker and indicated by arrow. Adjacent cells were digitally colored in red or green and named R and G. (**B**) Mitochondria were isolated from miR-965 or its antagomir-treated cells by centrifugation at 5800 × g for 5 min. The isolated mitochondria were exposed to recipient cells directly or through microfluidics for up to 5 h. The microfluidic mold made by pouring polydimethylsiloxane (PDMS) on cotton candy with a diameter of around 950 nm. (**C**) Transverse cross-sections of microfluidic ultra-structures using electron microscopy. (**D**) Transfer of mitochondria was visualized with mitotracker at 490 nm. Apoptosis (**E**) and caspase-3 (**F**) were determined using ELISA kit. (**G-J**) Proteins were extracted from MDA-MB-453 cells and expression of cytochrome C, Endo G, or Mcl-1 was determined using western blotting. Results are the means ± SE of 6 experiments in each group. *Significantly different from treatment of miRNA negative control (NC), *P* < 0.05. ^#^Significantly different from treatment of unsealed mitochondria following miR-965, *P* < 0.05

To examine the cellular properties in more depth, refractive index images were magnified using tomographic microscopy. Intriguingly, we detected tubular-shaped parts moving from one cell to another and mitotracker staining confirmed that these were mitochondria (Fig. 3A). Migration of mitochondria between cells has been reported to improve the viability or metabolism of the recipient cell [2, 9, 12]. We hypothesized that mitochondrial migration induces apoptosis in the recipient cell. To determine this, MDA-MB-453 cells were treated with miRNA negative control (NC), mja-miR-965, or antagomir-965 (Fig. 3B). In addition, we designed a microfluidic system to create an environment similar to nanotubes (Fig. 3B, Supplementary Fig. 1D). Considering that the diameter of the nanotube is 10–1000 nm and the mitochondrial diameter is approximately 500 nm, we devised a microfluidic system using polydimethylsiloxane (PDMS) which was poured into cotton candy to make a mold followed by addition of water to remove to remove the filaments. The microfluidic device was scanned with an electron microscope and the diameter measured to be about 950 nm (Fig. 3C). The isolated mitochondria were introduced into the microfluidic device at a flow rate of 10–30 μm/s. The mitochondria were stained with mitotracker which were shown to remain detectable up to 10 h in recipient tumor cells (Fig. 3D). Mitochondria that have passed through the microfluidic device are referred to herein as unsealed mitochondria (Fig. 3B). We next treated MDA-MB-453 cells with either the isolated or unsealed mitochondria. The isolated mitochondria or unsealed mitochondria did not lead to an increase in apoptosis, caspase-3, or cytochrome C in recipient cells (Fig. 3E-H). However, unsealed mitochondria extracted from miR-965-treated cells led to an increase in apoptosis of the recipient cells (Fig. 3E-H). Furthermore, regardless of miR-965 or antagomir treatment, unsealed mitochondria increased Endo G expression in recipient cells (Fig. 3I). Also both isolated and unsealed mitochondria extracted from miR-965-treated cells decreased Mcl-1 in recipient cells (Fig. 3J). Finally, antagomir treatment attenuated the miRNA-induced apoptotic genes (Fig. 3E-H), with the exception of Endo G (Fig. 3I).

### Endo G is essential for apoptosis through cellular transport of mitochondria

**Fig. 4:**
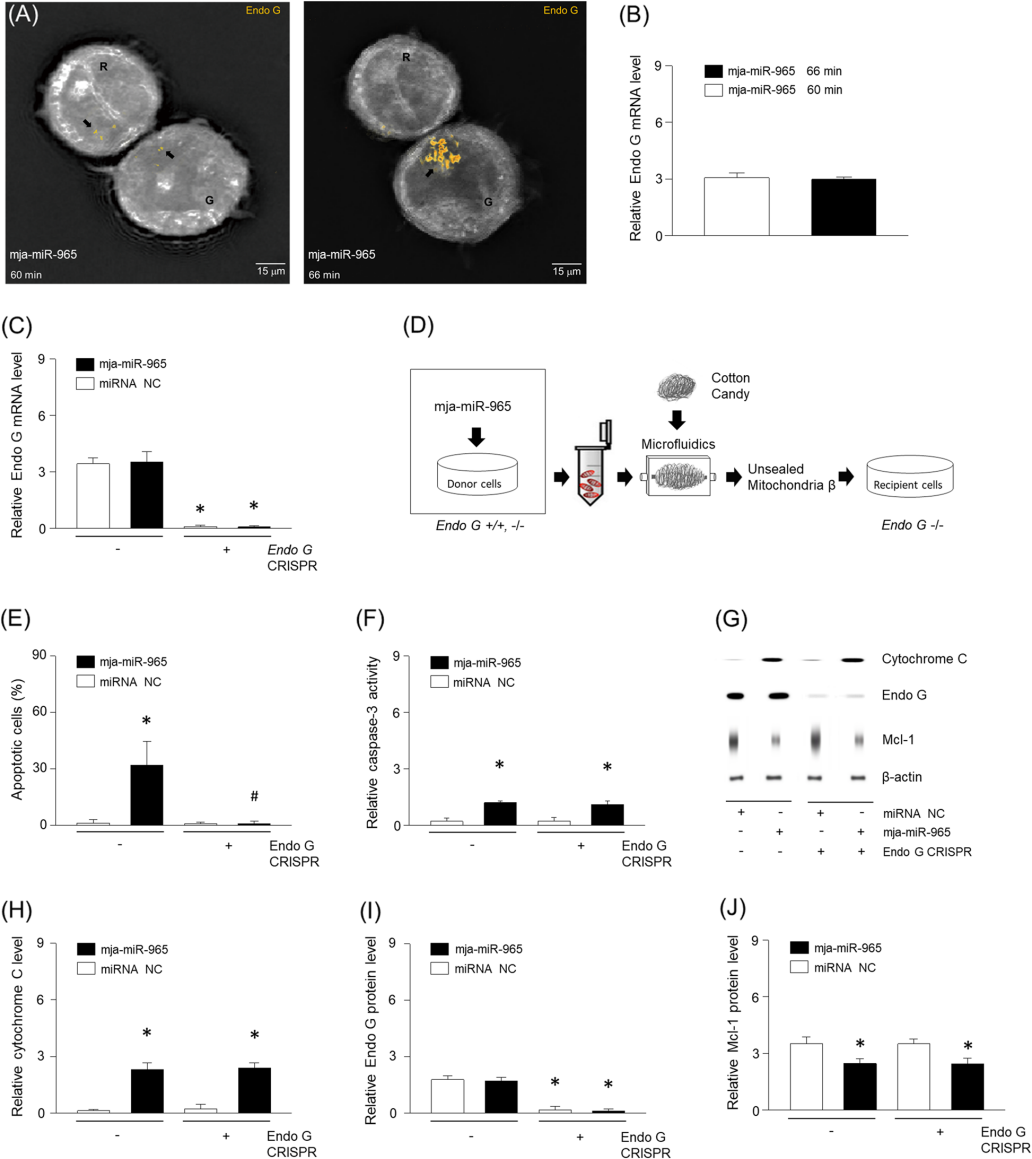
Mitochondria disgorged Endo G in narrow space. (**A**) Following treatment of miR-965, refractive index images of MDA-MB-453 were immunostained to detect Endo G (orange; indicated by arrow) at 60 or 66 min. Adjacent cells were digitally colored in red or green and named R and G. (**B**) *ENDOG* expression was measured using RT-PCR at 60 or 66 min. (**C**) Levels of Endo G miRNA in MDA-MB-453 treated with *ENDOG* CRISPR-plasmid ( +) or control CRISPR-plasmid (-). (**D**) Mitochondria isolated from Endo G knockout cells were passed through microfluidics into Endo G deficient MDA-MB-453. Following knock down of Endo G, apoptosis (**E**) and cleaved caspase-3 (**F**) were determined using ELISA kit and (**G-J**) the level of cytochrome C, Endo G, or Mcl-1 were determined using western blotting. Results are the means ± SE of 6 experiments in each group. *Significantly different from treatment of unsealed mitochondria following miRNA negative control (NC), *P* < 0.05. ^#^Significantly different from treatment of unsealed mitochondria from *Endo G* + / + cells, *P* < 0.05

We noted an accumulation of Endo G protein in the subcellular region proximal to the adjacent cell at 66 min post treatment with miR-965 (Fig. 4A, Supplementary Fig. 2A). This surge in protein levels was not associated with any differences in mRNA levels (Fig. 4B, Supplementary Fig. 2B-H). We posit that Endo G was released by the tunneled mitochondria. In order to better understand the effects of mitochondrial Endo G, *ENDOG* was knocked down using CRISPR-plasmid and confirmed using a real time-PCR (Fig. 4C). Following treatment with miR-965, mitochondria were isolated from MDA-MB-453 cells with or without *ENDOG* knockdown (Fig. 4D). *ENDOG* deficient cells were then treated with unsealed mitochondria referred herein as unsealed mitochondria β. Regardless of microRNA treatment, the unsealed mitochondria β from *ENDOG* deficient cells did not induce apoptosis (Fig. 4E). However, the unsealed mitochondria β from *ENDOG* deficient cells still increased caspase-3 and cytochrome C (Fig. 4F-H). In addition, the unsealed mitochondria β did not increase Endo G (Fig. 4I), but decreased Mcl-1 levels (Fig. 4J). Taken together the results indicate that disgorged Endo G through tunneling nanotubes is essential to promote tumor cell apoptosis.

### Unsealed mitochondria have a synergistic effect with doxorubicin

**Fig. 5:**
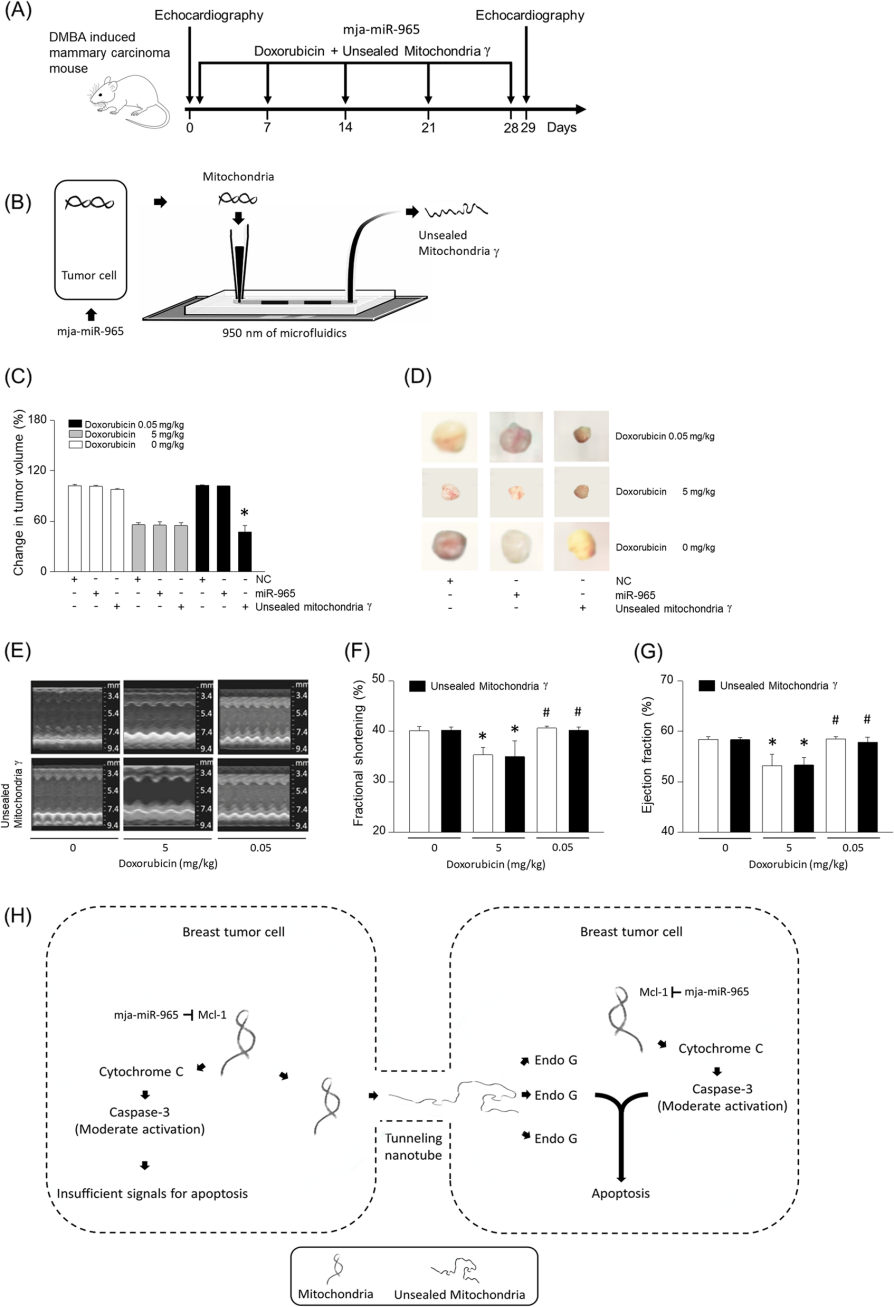
Effect of unsealed mitochondria in DMBA induced mammary carcinoma mouse. (**A**) Beginning at 5 weeks of age, female mice were given 1 mg of DMBA by oral gavage and 30 mg of subcutaneous pellets of MPA for 6 weeks. Mice with DMBA-induced mammary carcinoma were treated with doxorubicin or unsealed mitochondria γ. Doxorubicin was administered weekly by intraperitoneal injection (5 mg/kg or 0.05 mg/kg body weight) for 4 weeks. Unsealed mitochondria γ were administered weekly with 1 × 10^4^ per gram of body weight via the tail vein. The arrow indicates the injection days. (**B**) Schemata of the experimental setup to generate unsealed mitochondria γ using nano size-microfluidic device. (**C-D**) Bar graph or images of tumor volume change for mice treated with doxorubicin or unsealed mitochondria. (**E**) M-mode echocardiographic images from mice with DMBA-induced mammary carcinoma treated with doxorubicin or unsealed mitochondria. (**F-G**) Fractional shortening and ejection fraction were determined from the M-mode images. (**H**) Mitochondria from tunneling nanotube-released Endo G promoted breast tumor cell apoptosis with increased cleaved caspase-3. Results are the means ± SE of 6 experiments in each group. *Significantly different from treatment of 0 mg/kg doxorubicin, *P* < 0.05. ^#^Significantly different from treatment of 5 mg/kg doxorubicin, *P* < 0.05

In order to assess the lethal effects of unsealed mitochondria in vivo, mice with DMBA-induced mammary carcinoma were treated with miR-965, doxorubicin, or unsealed mitochondria (Fig. 5A). We refer to isolated mitochondria from the miR-965 treated cells that have passed through the microfluidics as unsealed mitochondria γ (Fig. 5B). 5 mg/kg of doxorubicin reduced the tumor volume, whereas 0.05 mg/kg of doxorubicin or miR-965 alone did not have any tumoritoxic effects (Fig. 5C). However, even at low concentrations of doxorubicin at 0.05 mg/kg, tumor growth was inhibited together when combined with unsealed mitochondria γ (Fig. 5C and D). In keeping with well-known cardiotoxic effects of doxorubicin, cardiac fractional shortening and ejection fraction decreased in the doxorubicin group at 5 mg/kg (Fig. 5E-G). However, 0.05 mg/kg of doxorubicin or the unsealed mitochondria γ did not lead to any adverse cardiac effects (Supplementary Fig. 1E and F). Overall, mitochondria through tunneling nanotubes can effectively synergize with subtoxic levels of doxorubicin to cause tumor cell death (Fig. 5H).

## Discussion

As the shell of crustaceans has been known to inhibit the growth of tumor, we carefully observed the shell structure of *Marsupenaeus japonicas* [25, 26]. In the shell membrane, a number of micro vesicles were found with predominance of mja-miR-965. We found that miR-965 was effective in inducing apoptosis of MDA-MB-453 cancer cells through transcellular migration of mitochondria via tunneling nanotubes.

While transcellular migration of mitochondria through tunneling nanotubes has been generally shown to impede apoptosis and optimize metabolism [3, 6, 9], we show in this study that this notion can also cause cytotoxic effects. Biggest challenge in this area of investigation has been with obtaining accurate imaging of tunneling nanotubes and mitochondria using optical microscopy. Difficulties around processing using chemicals and fixatives in addition to the limitations of two-dimensionality can contribute to the overall limitations of accurate assessment of tunneling nanotubes and mitochondria. Therefore we used tomographic microscopy which can provide the internal structure of cell images from multiple angles and render digitally reconstructed images according to the difference in refractive index, whereby tunneling nanotubes and mitochondria can be distinguished and visualized without special staining [14, 16]. Using such tomographic images, we reveal that mitochondria migrated to an adjacent cell through tunneling nanotubes to trigger apoptosis. Intriguingly, migration of mitochondria alone did not lead to apoptosis. Therefore, we hypothesized that an additional factor was needed for apoptosis to proceed.

It has been known that in addition to caspase-3, Endo G is critical for apoptosis [27]. Intriguingly, we found an increase in Endo G following passing of mitochondria through the tunneling nanotube. Since this was not associated with mRNA of Endo G, we posit that this accumulation of Endo G occurred as a result of release from transported mitochondria. To recapitulate this process, PDMS was poured onto cotton candy fibers with a diameter of about 950 nm to create a tunneling nanotube-like device. Mitochondria that have passed through the narrow nano-sized area which we referred to herein as unsealed mitochondria and the organelles from miR-965-treated cells induced apoptosis of breast tumor cells. In contrast, mitochondria of cells treated with its antagomir did not induce the tumor cell death. Moreover, unsealed mitochondria increased the amount of Endo G in recipient cancer cells regardless of miR-965. To confirm the relationship between mitochondria and Endo G, experiments were performed on breast tumor cells deficient in *ENDOG*. Mitochondrial delivery from miR-965-treated *ENDOG*-deficient cells did not induce cell death despite passing through a microfluidic system. In summary, miR-965 triggers effective tumor apoptosis by releasing Endo G in the recipient cells after transcellular migration of mitochondria.

To assess the role of this process in vivo, mice with DMBA-induced mammary carcinoma were administered with doxorubicin or unsealed mitochondria. While unsealed mitochondria from miR-965-alone did not have tumoricidal effects, it effectively synergized with subtoxic dose doxorubicin in reducing tumor size thereby sparing its well-known cardiotoxic effects. Although the mechanism of the unsealed mitochondria in the heart is unknown, reported studies suggest that Endo G is involved in cell survival rather than apoptosis of cardiomyocytes [28].

Taken together, we show that the mitochondria of microfluidics may provide novel strategies to effectively kill tumor cells. Lethal effects of mitochondria can potentially be harnessed using physical properties of delivery.

## Conclusion

In summary, our study revealed that shrimp miRNA could regulate the number of breast tumor cells. The mechanism was involved in lethal mitochondria through tunneling nanotubes. We reproduced the physical properties of delivery with microfluidics, which provide novel strategies to effectively kill tumor cells.

## Materials and methods

### Animal treatment

All mice were in C57BL/6 (RRID: 2,159,769) background and purchased from Charles River. All experiments using animals were approved and performed by the Ewha Womans University Animal Care Committee (Guide for the care and use of laboratory animals; IRB number: ESM14-0260). Forty female mice were each given weekly 1 mg doses of 7,12-Dimethylbenzathracene (DMBA) in 0.2 ml of sesame oil by oral gavage for six weeks and were implanted with 30 mg pellets of compressed medroxyprogesterone acetate (MPA) subcutaneously beginning at 5 weeks of age. Doxorubicin was administered weekly by intraperitoneal injection (5 mg/kg body weight, 300 μl injection volume) for four weeks. Mice were then maintained for an additional week.

### Cell culture

Human breast cancer cell lines, MDA-MB-453 (ATCC; RRID: CVCL_0418), was maintained in Dulbecco’s MEM (11,885, Gibco, USA) with 10% fetal bovine serum (16,000,044, Gibco, USA) at 37 °C under an atmosphere of 95% O_2_ and 5% CO_2_. Cells were exposed to miRNA negative control (MMIR-000-PA-1, System Biosciences), synthetic miR-965 or its antagomir (Bioneer, South Korea) using a kit from System Biosciences (EXFT10A-1, System Biosciences) for 5 h.

### Optical tomographic microscope

Green light (λ = 520 nm, exposure 0.2 mw/mm^2^) from a laser diode was split into cells and reference beam at Nanolive (3D cell explorer, Switzerland). Cells were illuminated with a laser beam inclined at 45° which rotated around the sample 360°. Holographic images were recorded on a digital camera by combining the beam that had passed through the cells with the reference beam. 3D cell images were recorded up to 30 μm depth of reconstruction. Mitochondria were visualized with MitoTracker (M7514, Thermo Fisher Scientific, USA) at 490 nm.

### Experimental procedures using microfluidic device

Cotton candy sheets were sealed with polydimethylsiloxane (PDMS) to construct microfluidic mold. After hardening, cotton candy fibers were removed by perfusing with water to make microfluidic mold of approximately 950 nm. Each microfluidic device was connected by polythene tubing (PE10, Braintree scientific, USA) with an inner diameter of 0.28 mm. Fluid flow was controlled by individual peristaltic pump (3,200,243, Dolomite, UK). Isolated mitochondria were introduced to microfluidic devices at a flow rate of 10–30 μm/s. After passing through the microfluidic channels, mitochondria were transported into cells.

### Analysis of RNA using real-time PCR

RNA levels of *ENDOG* (Hs00172770_m1) were determined using primer/probe set from Life Technology. Real-time PCR was performed with TaqMan universal PCR Master Mix on an ABI Real time PCR System 7000 (Applied Biosystems, USA). PCR conditions were 50 °C for 2 min and 95 °C for 10 min, followed by 40 cycles of 95 °C for 15 s and 60 °C for 1 min. For each experimental sample, the relative abundance value was normalized to the value derived from *ACTB* (Hs03023943_g1, Life Technology) as housekeeping control gene. Relative mRNA levels were quantified using the comparative 2^−ΔΔCT^ method. The extracted miRNA were determined using microarray service (Macrogen, South Korea).

### ELISA for detection of apoptosis or caspase-3 activity

Apoptosis was determined using ssDNA ELISA Kit (APT225, Sigma-Aldrich, USA). Briefly, cell plates were fixed and incubated with ABTS solution for 30 min to allow binding to HRP at 37 °C. To denature DNA, cell plates were incubated for 20 min at 75 °C. After cooling at 4 °C for 5 min, plates were blocked in 5% skim milk (70,166, Sigma-Aldrich) in PBS at 37 °C for 1 h. Cells were incubated with antisera mixture for 30 min and washed with PBS. After treatment with stop solution, absorbance was measured at 405 nm. Caspase-3 activity was determined using Cleaved Caspase-3 ELISA kit (ab220655, abcam, USA). Cells were fixed and incubated with antibody cocktail at 37 °C for 1 h, then washed and incubated with TMB solution for 30 min. After treatment with stop solution, absorbance was measured at 450 nm.

### Western blot

Cells were homogenized and centrifuged at 5,000 g for 20 min. Protein content of the supernatant was diluted, boiled with sample loading dye, and 100 mg were loaded in SDS-PAGE (4561033EDU, Bio-Rad). After blotting, membranes were blocked in 5% skim milk (70,166, Sigma-Aldrich) in PBS containing 0.1% Tween-20 (P1379, Sigma-Aldrich). Membranes were incubated with antisera directed against cytochrome C (1:1000; #11,940, RRID: AB_2637071, Cell signaling technology, USA), Endo G (1:1000; #4969, RRID: AB_2098768, Cell signaling technology, USA), Mcl-1 (1:1000; ab28147, RRID: AB_776246, abcam, USA), or β-actin (1:1000; sc-47778, RRID: AB_626632, Santacruz Biotechnology, USA), then with secondary antibodies (mouse-specific HRP-conjugated antibody or rabbit-specific HRP-conjugated antibody). Bands were visualized using ECL (32,106, Thermo Scientific) detection kit and quantified by densitometry.

### Mitochondrial isolation and transfer

Cells were harvested from culture dishes with homogenization buffer (20 mM HEPES–KOH, 220 mM mannitol, and 70 mM sucrose) containing a protease inhibitor mixture (Sigma-Aldrich) and centrifuged at 2300 × g for 5 min. The cell pellet was resuspended with homogenization buffer and incubated on ice for 5 min at 4 °C. Cells were ruptured by 10 strokes using a 27-gauge needle. The homogenate was centrifuged at 5800 × g for 5 min, and mitochondria were harvested. The amount of isolated mitochondria was expressed as protein concentration by using the Bio-Rad protein assay kit (Bio-Rad, Richmond, USA). Mitochondrial transfer was conducted by co-incubating isolated mitochondria with cells (1 × 10^5^ cells/well of a 6-well plate) at 37 °C under 5% CO_2_ for up to 10 h. For in vivo experiments, mice were administered weekly with 1 × 10^4^ isolated mitochondria per gram of body weight via the tail vein.

### Transmission electron microscopy

We fixed the tissues of *Marsupenaeus japonicas* or mold of cotton candy-microfluidic with 3% buffered glutaraldehyde (G5882, Sigma-Aldrich) for 2 h and processed into resin (02,334, Polysciences, German). After embedding, the resin block was thin-sectioned by ultramicrotomy. Sections of 50–70 nm thickness were collected on metal mesh and stained with electron dense particles before imaging of ultrastructures, using the transmission electron microscope (H-7650, Hitachi-Science & Technology, Japan).

### CRISPR-mediated gene deletion

Clustered regularly interspaced short palindromic repeats (CRISPR) transfection of *ENDOG* in MDA-MB-453 was performed using a kit from Santa Cruz (sc-395739, Santacruz Biotechnology, USA). Briefly, in six-well culture plates, 10^6^ cells were plated and exposed to the *ENDOG* plasmid (sc-403263, Santacruz Biotechnology, USA) or negative control-CRISPR plasmid (sc-418922, Santacruz Biotechnology, USA) solution for 8 h at 37 ℃ in a CO_2_ incubator. Then, media was changed to Dulbecco’s MEM with 10% fetal bovine serum and incubated for another 18 h. The *ENDOG* expression was determined using RT-PCR.

### Echocardiographic assessment

For echocardiography, Vevo 2100 was used at Cardiovascular Research Center in Seoul. Mice were anesthetized with 2% isoflurane and maintained with 1.5% isoflurane followed by application of depilatory cream to the chest and wiped clean to remove all hair in the area of interest. The scanning probe (20 MHz) was used to obtain 2D images of the parasternal long axis. These 2D images were converted to M-mode.

### Statistical analysis

Values were means ± SE. The significance of differences was determined by a two-way analysis of variance (ANOVA), or a one way ANOVA followed by a Bonferroni post-hoc analysis where appropriate. Differences were considered significant when *P* < 0.05.

## Supplementary Information

**Additional file 1: Supplementary Figure 1.** Morphology and apoptosis in breast tumor cells. (A) Using tomographic microscopy, the number of filopodia was measured in primary epithelial breast tumor cells and MDA-MB-453. (B and C) Viability and morphology of MDA-MB-453 were observed for 48 h. (D) Efficiency of stained mitochondria uptake into MDA-MB-453. (E) Comparison of unsealed mitochondria derived from DMBA-induced mammary carcinoma. (F) Role of unsealed mitochondria on apoptosis in cardiomyocytes and breast epithelial cells. Results are the means ± SE of 6 experiments in each group. *Significantly different from treatment of Rapamycin and Y27632 for 0 h, *P* < 0.05. ^#^Significantly different from unsealed mitochondria on isolated cardiomyocyte, *P* < 0.05.

**Additiona file 2: Supplementary Figure 2.** The relationship between Endo G and apoptosis. (A) Following treatment of miR-965, refractive index images of MDA-MB-453 were immunostained to detect Endo G (orange) or mitochondria (green). Adjacent cells were digitally colored in red or green and named R and G. (B-E) Isolated mitochondria or unsealed mitochondria were produced in large quantities from approximately 6 X 10^8^ MDA-MB-453 cells. After precipitating mitochondria with a centrifuge, the supernatant of these samples were lyophilized and western blotting was performed for Endo G detection. (E-H) Transfection of Endo G ORF lentiviral particles (Origene, RC205089L1V) into MDA-MB-453 cells did not induce apoptosis. Results are the means ± SE of 6 experiments in each group. *Significantly different from treatment of isolated mitochondria, *P* < 0.05. ^#^Significantly different from treatment of miRNA negative control, *P* < 0.05.

## Data Availability

All data supporting the findings of this study are available within the paper.
