# Supplementary figures and images for: Shrimp miR-965 transfers tumoricidal mitochondria
Source: Biol Proced Online. 2022 Oct 26;24:16. doi: 10.1186/s12575-022-00178-8 (PMC9598032; doi:10.1186/s12575-022-00178-8)

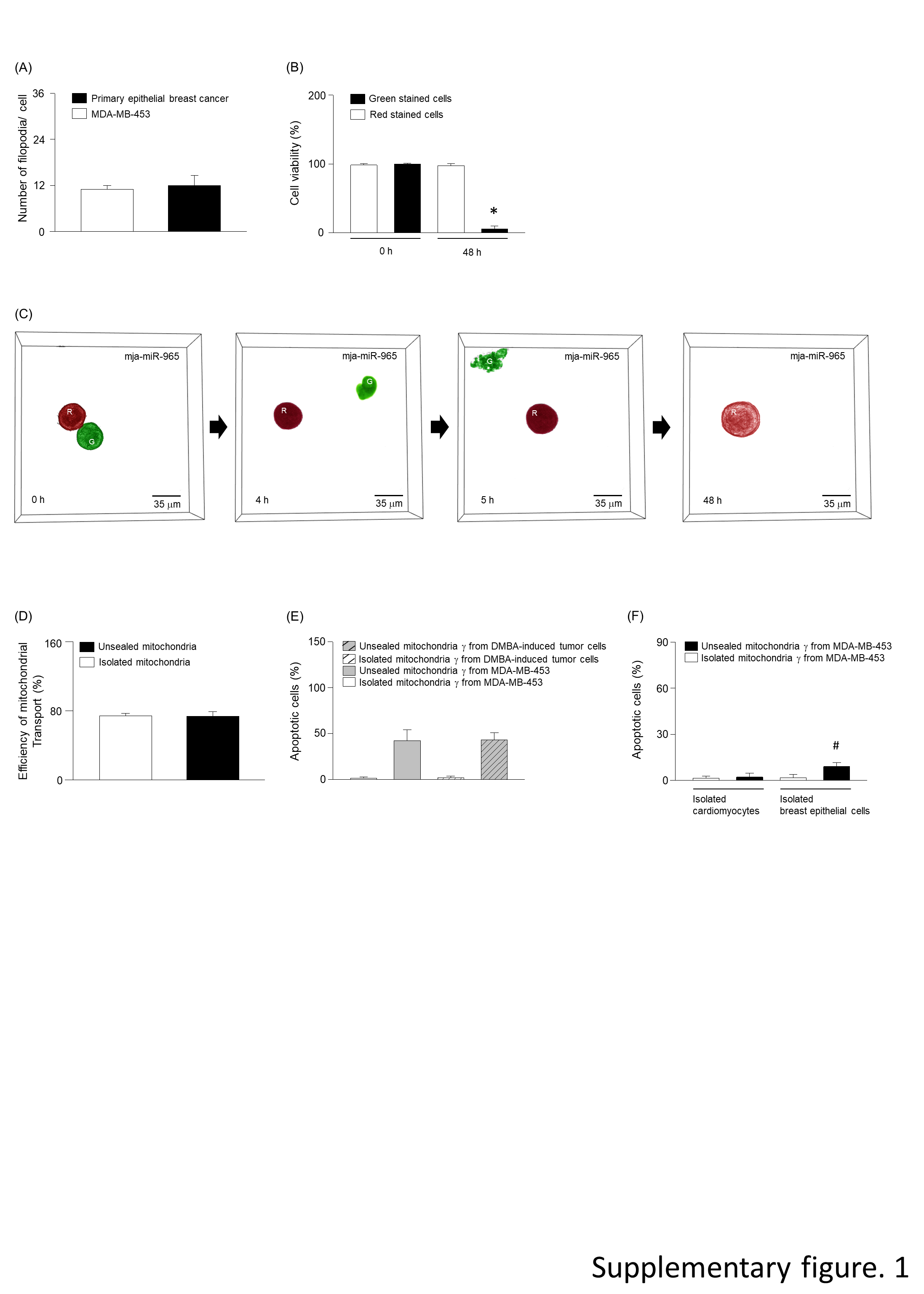

Supplement: Supplementary file 1 — Additional file 1: Supplementary Figure 1. Morphology and apoptosis in breast tumor cells. (A) Using tomographic microscopy, the number of filopodia was measured in primary epithelial breast tumor cells and MDA-MB-453. (B and C) Viability and morphology of MDA-MB-453 were observed for 48 h. (D) Efficiency of stained mitochondria uptake into MDA-MB-453. (E) Comparison of unsealed mitochondria derived from DMBA-induced mammary carcinoma. (F) Role of unsealed mitochondria on apoptosis in cardiomyocytes and breast epithelial cells. Results are the means ± SE of 6 experiments in each group. *Significantly different from treatment of Rapamycin and Y27632 for 0 h, P < 0.05. #Significantly different from unsealed mitochondria on isolated cardiomyocyte, P < 0.05. [file 12575_2022_178_MOESM1_ESM.tif]

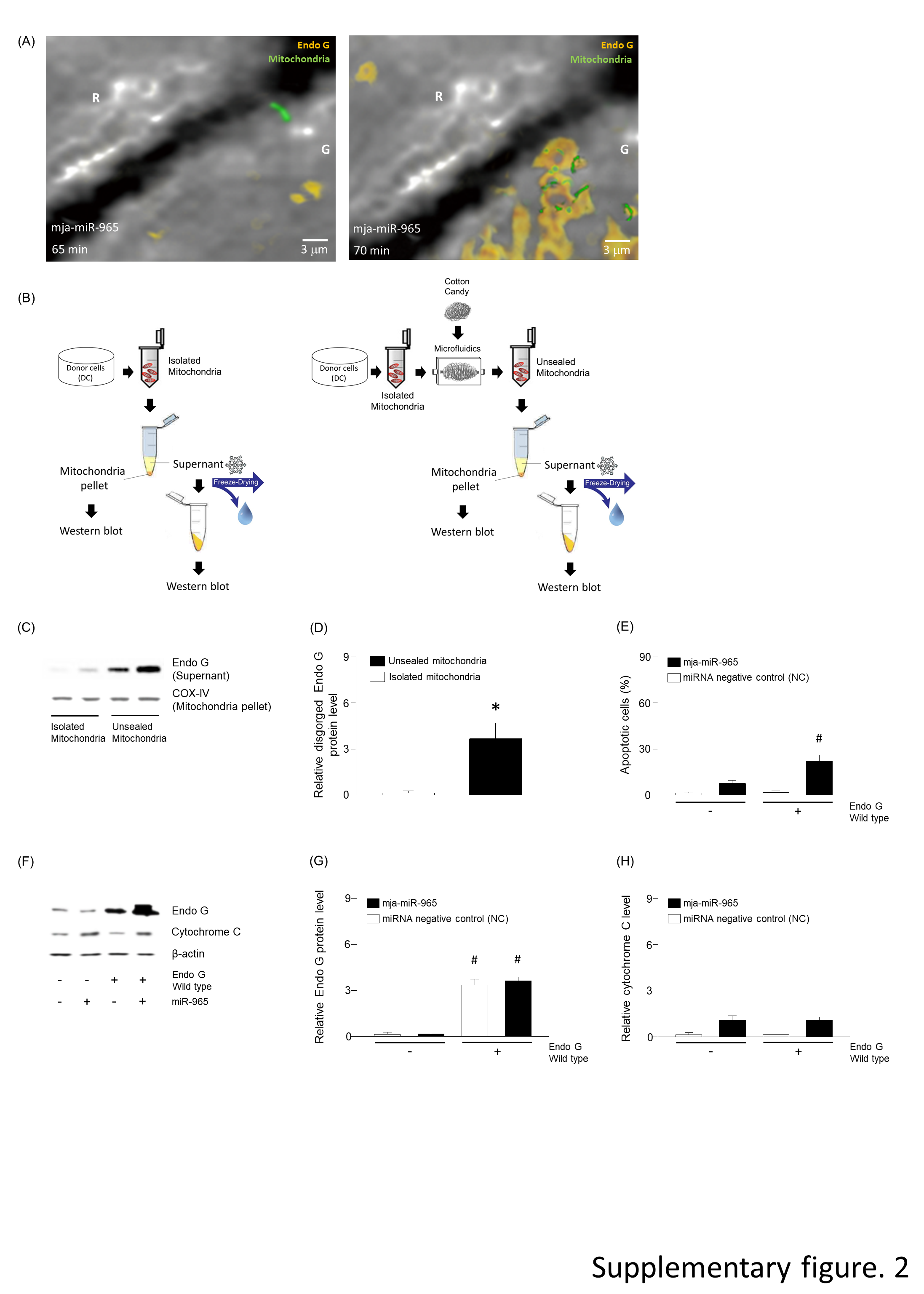

Supplement: Supplementary file 2 — Additiona file 2: Supplementary Figure 2. The relationship between Endo G and apoptosis. (A) Following treatment of miR-965, refractive index images of MDA-MB-453 were immunostained to detect Endo G (orange) or mitochondria (green). Adjacent cells were digitally colored in red or green and named R and G. (B-E) Isolated mitochondria or unsealed mitochondria were produced in large quantities from approximately 6 X 108 MDA-MB-453 cells. After precipitating mitochondria with a centrifuge, the supernatant of these samples were lyophilized and western blotting was performed for Endo G detection. (E-H) Transfection of Endo G ORF lentiviral particles (Origene, RC205089L1V) into MDA-MB-453 cells did not induce apoptosis. Results are the means ± SE of 6 experiments in each group. *Significantly different from treatment of isolated mitochondria, P < 0.05. #Significantly different from treatment of miRNA negative control, P < 0.05. [file 12575_2022_178_MOESM2_ESM.tif]
